# Self at Risk: Self-Esteem and Quality of Life in Cancer Patients Undergoing Surgical Treatment and Experiencing Bodily Deformities

**DOI:** 10.3390/healthcare11152203

**Published:** 2023-08-04

**Authors:** Ewa Wojtyna, Małgorzata Pasek, Aleksandra Nowakowska, Anna Goździalska, Małgorzata Jochymek

**Affiliations:** 1 Institute of Psychology, University of Silesia in Katowice, 40-007 Katowice, Poland; 2 Department of Nursing, Faculty of Health, University of Applied Sciences in Tarnów, 33-100 Tarnów, Poland; 3 Faculty of Health and Medical Studies, Andrzej Frycz Modrzewski Krakow University, 30-705 Kraków, Poland

**Keywords:** self-esteem, quality of life, cancer, implicit self-esteem, surgery

## Abstract

Self-esteem is an important factor determining QoL after surgical procedures leading to bodily deformities associated with cancer treatment. However, there are few data on which components of self-esteem are most closely related to QoL. The article presents two studies that aim to fill this gap. Study 1 concerns changes in global self-esteem and QoL in patients treated surgically for oral cancer (*n* = 35); Study 2 concerns changes in explicit and implicit self-esteem and QoL in women with breast cancer undergoing mastectomy (*n* = 96). The study was longitudinal with two measurements: before and after surgery. Both studies used the EORTC QLQ-C30 and Rosenberg’s SES questionnaires. In Study 2, the Implicit Association Test (IAT) was additionally performed. The patients’ global QoL and self-esteem deteriorated after surgery. In Study 1, patients with higher initial self-esteem showed a greater range of decreased self-esteem and QoL than patients with initially low self-esteem. In Study 2, the largest decreases in various dimensions of QoL and explicit self-esteem were observed in women with fragile self-esteem. A group of women with high explicit and implicit self-esteem showed the best QoL after mastectomy. Cancer patients with high, fragile self-esteem are at risk of the greatest deterioration in QoL and self-image after cancer surgery. These people should be given special psycho-oncological care.

## 1. Introduction

Cancer has a significant impact on a patient’s functioning and self-image [1]. While oncological treatment brings progressively better results and increases the patient’s chance of recovery or prolongation of life, their quality of life (QoL) remains a challenge [2]. This may particularly apply to people in whom the disease and/or its treatment causes visible changes in appearance or is the reason for deteriorated physical functioning [3,4,5].

Health-related QoL is a multidimensional construct located in the area of patient-reported outcomes (PROs), and concerns not only somatic, but also mental, spiritual, social and financial functioning [6,7,8,9]. Currently, many clinical decisions involve consideration of their impacts on the patient’s QoL [10]. Hence, a better understanding of the factors determining QoL becomes an important area of psycho-oncology and clinical oncology.

Self-esteem is one of the factors that are related to QoL and coping with the disease. Self-esteem refers to a subjective assessment of one’s worth, value and overall sense of oneself, including abilities and personal qualities [11,12,13,14]. It has frequently been shown that cancer lowers patients’ self-esteem [11,12,13]. However, for the psycho-oncological care of cancer patients, the reverse direction of this relationship is also important, that is, how self-esteem affects their QoL during oncological treatment.

Self-esteem is the basic cognitive structure of personality and is one of the most important motivational forces in human life [14]. It is developed during the life of an individual based on self-observation and feedback obtained from people in a close environment [15]. Self-esteem has a significant impact on emotions and determines certain actions and their evaluation. Thus, it plays an important role in the process of experiencing and coping with the disease and its treatment. Self-esteem affects the assessment of a stressful situation—the better the self-esteem, the more often the patient treats the disease as a challenge. This leads to more frequent positive emotions, such as hope [16], positive cognitive reframing of the illness and finally, constructive adaptation to the state of being ill [12,17]. The more positive someone’s self-esteem, the lower the likelihood of mental disorders (anxiety, depression) occurring in the course of the disease and during treatment [18,19]. Self-esteem is significantly related to the sense of control over the disease [20].

In conclusion, self-esteem is an important variable for assessing the disease situation, adaptation to the disease and the ability to cope with it. It is also an important factor related to the QoL of patients [21,22,23]. The above data indicate that research on self-esteem has important practical implications. Improving self-esteem can lead to coping better with the disease [22,24,25]. However, there is still little information on how self-esteem and QoL shape each other in a short period, including the period of adaptation after surgery, and how it significantly affects the appearance and functioning of cancer patients. In addition, there are few data on which components of self-esteem are most closely related to QoL. This article presents two studies that aim to fill this gap. The first concerns changes in global self-esteem and QoL in patients treated surgically for oral cancer; the second concerns changes in explicit and implicit self-esteem and QoL in women with breast cancer undergoing mastectomy.

## 2. Study 1: Self-Esteem and QoL in Patients with Oral Cancer

### 2.1. Introduction

Oral cancers are among the most common neoplasms in the head and neck area [26]. The QoL of patients with oral cancer is an important area taken into account in highly specialised patient care [27]. The mere occurrence of cancer in the oral cavity as well as the treatment undertaken may make communication difficult and cause eating and breathing problems, disorders in the senses of taste and smell and facial deformities, which often lead to social isolation, a sense of exclusion and significant difficulties in the patient’s daily functioning. All these factors cause a significant reduction in the patient’s QoL [28].

Surgical treatment of neoplastic lesions within the mandibular bones, jaws, tongue, the floor of the mouth or lips is a mutilating procedure that causes significant distortions in the lower part of the face. They cause disorders in the area of mandibular and maxillary occlusion, adduction, abduction and deviation of the mandible. There is also pain in the oral cavity, the shallow floor of the mouth, scarring, decreased mobility of the tongue, difficulties with speech and swallowing and thus, significant weight loss, cachexia and deterioration of physical fitness, which contribute to a decrease in QoL and self-esteem after surgical treatment [28,29,30]. Patients after resection of the tongue struggle with difficulty chewing and swallowing food, loss of taste and smell as well as with the flow of saliva and food from the mouth. Patients take longer to eat their meals, food regurgitates into the mouth or nose or the peristalsis of the upper digestive tract slows down.

The aim of Study 1 was (1) to assess self-esteem and QoL in patients with oral cancer before and after surgery, and (2) to determine the relationship between self-esteem and QoL in this group of patients.

### 2.2. Materials and Methods

#### 2.2.1. Study Design

The study was longitudinal and used a questionnaire. It was carried out in the Department of Maxillofacial Surgery at a Specialist Hospital in Krakow, Poland. The study received an endorsement from the Scientific Board of the University of Silesia in Katowice and was conducted in accordance with the principles of the Declaration of Helsinki. All obtained data were pseudonymised, which enabled the combination of pre- and post-test data while maintaining the principle of non-identification of patients.

The study included two measurements: a pre-test on the day before the surgical removal of a tumour and a post-test two weeks after surgery.

#### 2.2.2. Research Methods

The study used a questionnaire enabling the collection of basic socio-demographic and disease-related data.

The EORTC QLQ-C30 questionnaire together with the H&N35 module on head and neck cancers was used to assess health-related QoL.

EORTC QLQ-C30 is a tool that allows the assessment of QoL understood as the subjective perception of positive and negative aspects of a cancer patient’s symptoms, including physical, emotional, social and cognitive functions and, importantly, symptoms of the disease and the side effects of treatment [31]. This 30-item QoL questionnaire consists of a global scale of health and QoL, five functional scales (concerning the cognitive, physical, role-related, emotional and social spheres), three symptom scales (pain, fatigue and nausea and vomiting), single items examining other common symptoms (including sleep disorders, constipation, diarrhoea, loss of appetite and shortness of breath) and one item regarding the perceived impact of cancer and its treatment on the patient’s economic sphere. In the case of functional and symptom scales, a four-point Likert scale was used (answers from 1: ‘not at all’ to 4: ‘very much’), and in the case of two global health and QoL scales, a seven-point linear analogue scale is used. All scores are converted to a scale of 0–100. For functional and global QoL items, higher scores indicate better functionality and QoL (favourable conditions). In the case of symptom scales, higher scores mean greater symptomatology (unfavourable conditions).

EORTC QLQ-H&N35 is a 35-item QoL scale for head and neck cancer patients [32]. H&N35 consists of seven scales assessing pain, swallowing, senses (taste and smell), speech, social eating, social contacts and sexuality. In addition, 11 individual items for various symptoms are included. Items 1–30 are rated on a four-point Likert scale. Items 31–35 are in the no/yes format. The scores are converted to a scale of 0–100, with a higher score indicating more unfavourable conditions for all items.

The Cronbach’s alpha reliability index for the scales included in the EORTC tool ranges from 0.75 to 0.98.

The Rosenberg Self-esteem Scale (SES), translated to Polish, was used to assess self-esteem [33]. The scale is a one-dimensional tool that allows the assessment of general self-esteem—a relatively constant disposition understood as a conscious attitude (positive or negative) towards the self. It consists of ten diagnostic statements. A patient was asked to indicate on a four-point scale to what extent he or she agrees with each of these statements. On the SES, the tested person can score from 10 to 40, with higher scores indicating better self-esteem. The Cronbach’s alpha reliability index for this scale ranges from 0.72 to 0.87.

#### 2.2.3. Study Participants

The studied group included 35 patients diagnosed with cancer in the oral cavity who qualified for surgical treatment. The sample size was calculated using G*Power 3.1 software, assuming a statistical power of 0.85 and an effect size of 0.5. With the assumption of approximately 10% dropout, the sample size was calculated at *n* = 35. The criteria for inclusion in the study were informed consent to participate in the study, diagnosis of head and neck cancer eligible for surgical treatment as well as a mental state enabling understanding and completing the questionnaires independently. The criteria for exclusion from participation in the study were a lack or withdrawal of consent to participate in the study, an acute crisis caused by factors other than cancer, active addiction and psychotic symptoms.

The characteristics of respondents are presented in Table 1.

#### 2.2.4. Statistical Analysis

Statistical analysis of the data was performed using the SPSS 24.0 statistical package (IBM Corp., Armonk, NY, USA). Normal distribution was tested with the Kolmogorov–Smirnov test. If data were not normally distributed, they were transformed using log transformation. A dependent t test was performed to verify differences between pre- and posttest outcomes. A repeated-measurement analysis of variance with “group” (high and low self-esteem) and “time” (pretest and posttest) and the interaction between “group” and “time” were performed to investigate the effect of surgery on QoL among patients with oral cancer. Bonferroni-corrected post hoc tests were performed, as required. Correlation analysis between self-esteem and QoL were performed with Spearman or Pearson correlation (two-sided). For all the above analyses, *p*-values of less than 0.05 were considered significant.

### 2.3. Results

The study included 35 people diagnosed with oral cancer, aged 28 to 80 (M = 54.40; SD = 15.13), of which 57.1% were women.

The patients’ global QoL and self-esteem deteriorated after surgery (Table 2). In individual areas of QoL, deterioration was observed in the following areas: physical functioning, role functioning, fatigue, nausea and vomiting, pain, dyspnoea, insomnia, constipation, swallowing, opening mouth, dry mouth, sticky saliva, senses problems, feeling ill, speech problems, trouble with social contact, trouble with social eating, reduced interest in sex, pain killers, nutritional supplements, feeding tube and weight loss. 

Before surgery, self-esteem was correlated with QoL in the following areas: global health status/QoL (*rho* = 0.33; *p* = 0.041), physical functioning (*rho* = 0.30; *p* = 0.049), emotional functioning (*rho* = 0.32; *p* = 0.044), insomnia (*rho* = −0.31; *p* = 0.047), trouble with social contact (*rho* = −0.34; *p* = 0.040), reduced interest in sex (*rho* = −0.48; *p* = 0.003) and the use of pain killers (*rho* = −0.35; *p* = 0.039). In turn, after the procedure, self-esteem was associated with higher QoL in the following areas: physical functioning (*rho* = 0.40; *p* = 0.018), nausea and vomiting (*rho* = −0.47; *p* = 0.005), appetite loss (*rho* = −0.36; *p* = 0.033), financial difficulties (*rho* = −0.33; *p* = 0.032), trouble with social contact (*rho* = −0.33; *p* = 0.031), sexual problems (reduced sexuality scale, *rho* = −0.36; *p* = 0.030) and feeding tube problems (*rho* = −0.42; *p* = 0.012). It is worth noting that self-esteem before surgery was positively correlated with QoL after surgery in the following areas: emotional (*rho* = 0.31; *p* = 0.048) and cognitive functioning (*rho* = 0.33; *p* = 0.032).

**Figure 1:**
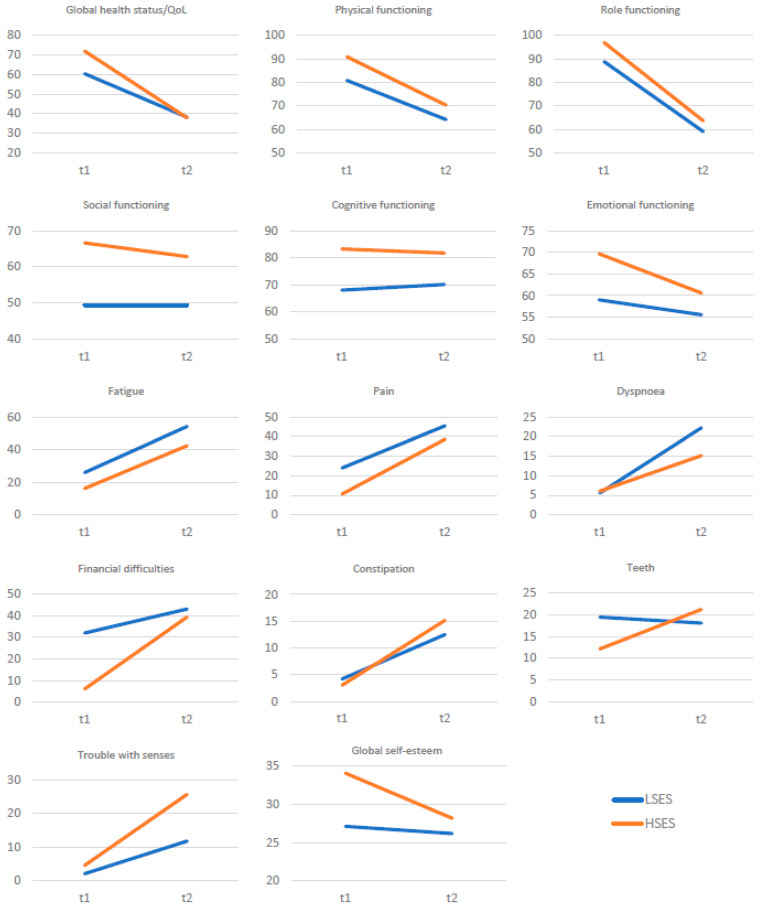
QoL (EORTC QLQ-C30) and self-esteem (SES) before and after surgery among patients with oral cancer and low (LSES) or high (HSES) baseline self-esteem (only statistically significant results are presented).

Depending on the initial level of self-esteem, different dynamics of change were observed before and after the procedure in the examined variables (Figure 1). Patients with higher initial self-esteem showed a greater range of decreased self-esteem, global QoL and emotional and social functioning after the procedure than patients with initially low self-esteem.

Compared to patients with low self-esteem, those with initially high self-esteem showed a greater increase in difficulties in terms of financial problems, constipation, pain and problems with the teeth and senses. In QoL dimensions such as fatigue, dyspnoea, physical functioning, role functioning and cognitive functioning, patients with initially high self-esteem functioned better after the procedure than those with low self-esteem.

### 2.4. Discussion

The results of Study 1 showed a reduction in QoL and self-esteem after surgical treatment in patients with oral cancer. A positive correlation was found between self-esteem and various dimensions of health-related QoL, which remains consistent with the reports of other researchers [5]. However, our study also suggested different processes of change in the studied variables before and after the procedure in people with initially high and low self-esteem.

Patients with initially high self-esteem experienced stronger decreases in self-esteem and QoL in many dimensions than those with initially low self-esteem. This observation seems surprising in the light of reports on the protective role of self-esteem in relation to QoL, resilience and coping with stress and illness in cancer patients [20,21,22,34,35]. It may be the result of the small size and high heterogeneity of the compared groups with low and high self-esteem. Nevertheless, it is worth remembering that self-esteem is not a uniform construct [36]. In this study, the SES was used to examine so-called global self-esteem as a general, subjective, cognitive and explicit self-image [37]. Meanwhile, Baumeister et al. [38] have shown that among people with high declarative self-esteem, there are those with stable and fragile self-esteem. These two types of self-esteem are related to the concept of implicit attitudes. Implicit self-esteem belongs to the experiential system and thus is automatic, non-verbal, associative and strongly related to affect. In contrast to explicit self-esteem, it is less distorted by self-presentation mechanisms [39], but it is more susceptible to experimental manipulation and less stable over time than its explicit counterpart. Fragile self-esteem is a situation in which high explicit self-esteem is accompanied by low implicit self-esteem [40,41]. It is possible that in the studied group, there were people with fragile self-esteem and the situation of the surgery influenced the change in the implicit level, which translated into worse functioning and its perception by these people after surgery.

## 3. Study 2: Explicit and Implicit Self-Esteem and Quality of Life in Women with Breast Cancer

### 3.1. Introduction

Mastectomy in women with breast cancer is a frequent method of treatment, but, at the same time, it is a risk factor in patients’ self-image deterioration. It has been repeatedly shown that both the disease itself and its treatment lower the self-esteem and QoL of patients [42].

The results of Study 1 showed that oral cancer patients with high self-esteem experienced particularly strong decreases in self-esteem and QoL after surgery. In Study 2, it was decided to take a closer look at this issue in a group of patients characterised by greater homogeneity, and to examine the self-esteem itself both explicitly and implicitly.

Explicit self-esteem is defined as a general assessment of self-worth [14,43]. Negative or low self-esteem is a non-specific risk factor for mental health and is associated with a wide spectrum of psychopathological symptoms [44,45,46,47]. Two-process models of self-esteem propose that in addition to the explicit, purposeful cognitive process of self-evaluation, there is also an impulsive or more ‘automatic’ process [48], referred to as ‘implicit self-esteem’. This type of self-esteem it is also defined as global self-esteem that people are unable or unwilling to report [48]. Although explicit and implicit types of self-esteem are thought to influence each other, they show little correlation [49] and are therefore viewed as two different self-esteem constructs.

In a situation where cognitive resources are limited or time constraints exist or purposeful reflection is impossible, this implicit self-esteem is shaped by the automatic processing of affective experiences [36,47,50]. Measuring both implicit and explicit types of self-esteem makes it possible to look additionally at the possible discrepancy between them. Disparities in either direction are maladaptive, as they represent insufficient self-presentation integration and are associated with more negative mental health outcomes than corresponding low or high implicit and explicit self-esteem [51].

The aim of Study 2 was to examine changes in QoL and self-esteem in women with breast cancer who underwent a mastectomy, depending on their initial explicit and implicit self-esteem.

### 3.2. Materials and Methods

#### 3.2.1. Study Design

The study was longitudinal. It received a positive response from the Scientific Research Committee of the University of Silesia in Katowice. All collected data have been pseudonymised. The study included two measurements: a pre-test carried out seven days before mastectomy and a post-test conducted two months after the procedure.

#### 3.2.2. Research Methods

The study was conducted using a questionnaire enabling the collection of basic socio-demographic and disease-related data.

The EORTC QLQ-C30 [29] questionnaire was used to assess health-related QoL, and the SES [31] was applied to examine explicit global self-esteem.

The Implicit Association Test (IAT) was used to assess implicit self-esteem [39,52,53,54]. A patient performs this test on a computer. The person is asked to assign specific words to given categories (combinations of two pairs of categories are used: Self and Non-Self, as well as Positive and Negative). The time taken by the person to assign the words appearing on the screen to specific categories allows the calculation of the so-called d-score, which ranges from −2 to 2 [52]. The higher the score, the higher the implicit self-esteem. The IAT was carried out in the Inquisit 5 Lab software, which uses the improved algorithm described by Greenwald et al. [52] to calculate this indicator. To carry out further analyses, the indicators of implicit and explicit self-esteem were standardised, making it possible to determine the discrepancy between the variables, according to the procedure described by Creemers et al. [41].

In the pre-test, all the above tools were used, while in the post-test, the patients completed two questionnaires: SES and EORTC QLQ-C30.

#### 3.2.3. Study Participants

The studied group consisted of 96 women diagnosed with breast cancer who qualified for surgical treatment (mastectomy). The criteria for inclusion in the study were informed consent to participate in the study, diagnosis of breast cancer, qualification for mastectomy, and age (18 to 60), as well as a mental state enabling understanding and completing the questionnaires independently. The criteria for exclusion from participation in the study were a lack or withdrawal of consent to participate in the study, an acute crisis caused by factors other than cancer, pregnancy, active addiction and psychotic symptoms. The study participants were recruited using an application form distributed by the coordinator in the oncology wards of Silesian hospitals. The study was conducted in a laboratory in the Institute of Psychology of the University of Silesia.

The characteristics of the respondents are presented in Table 3.

#### 3.2.4. Statistical Analysis

A statistical analysis of the data was performed using the SPSS 24.0 statistical package IBM Corp., Armonk, NY, USA). Normal distribution was tested with the Kolmogorov–Smirnov test. If data were not normally distributed, they were transformed using log transformation. For parametric, normally distributed data, an independent t test was performed to verify group differences, whereas for nonparametric data, the Mann–Whitney U test was used. A repeated-measurement analysis of variance with “group” (high explicit and implicit self-esteem, fragile self-esteem and low explicit and implicit self-esteem) and “time” (pretest and posttest) and the interaction between “group” and “time” were performed to investigate the effect of mastectomy on QoL. Bonferroni-corrected post hoc tests were performed, as required. Correlation analysis between self-esteem and QoL were performed with the Spearman or Pearson correlation (two-sided). For all the above analyses, *p*-values less than 0.05 were considered significant.

### 3.3. Results

The study included 96 women with breast cancer aged 34 to 56 (M = 51.76; SD = 6.83).

Based on the results obtained in the SES, groups with low (SES score < M − 0.5 SE), moderate and high (SES score > M + 0.5 SE) explicit self-esteem were distinguished. Based on the estimated *d*-score (IAT), the study participants were classified into three groups: high implicit self-esteem (*d* > +0.5), low implicit self-esteem (*d* < −0.5) and a group with undifferentiated implicit self-esteem (*d* ranging from −0.5 to +0.5). The joint consideration of explicit and implicit self-esteem results allowed us to distinguish the following groups: high explicit and implicit self-esteem (HSES), fragile self-esteem and low explicit and implicit self-esteem (LSES). In the studied group, 21 people were characterised by explicit low self-esteem, and among women with moderate or high explicit self-esteem, 43 showed fragile self-esteem. Moreover, 32 people showed high implicit self-esteem (Table 3).

After mastectomy, the analysis for the whole group showed a decrease in explicit self-esteem and QoL in the following dimensions: global health status/QoL, physical, role, emotional and social functioning, fatigue, insomnia, financial difficulties and pain (Table 4).

**Figure 2:**
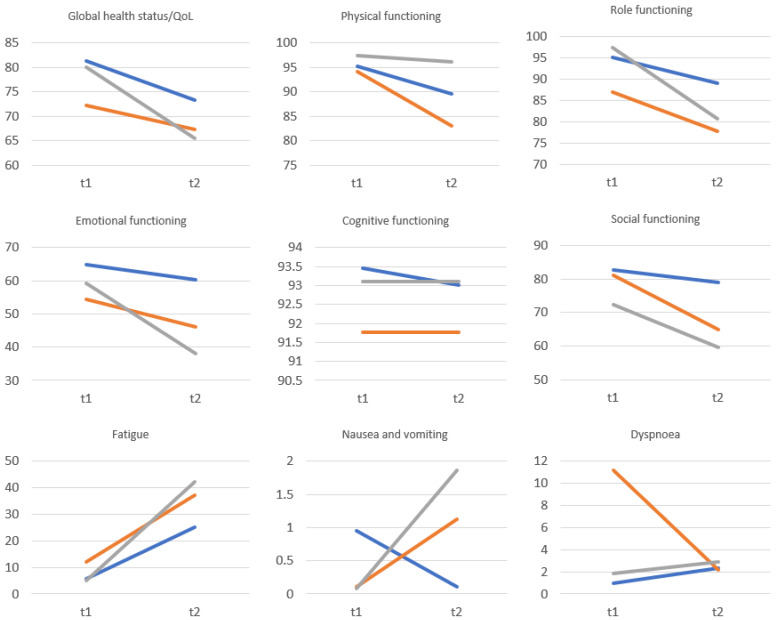

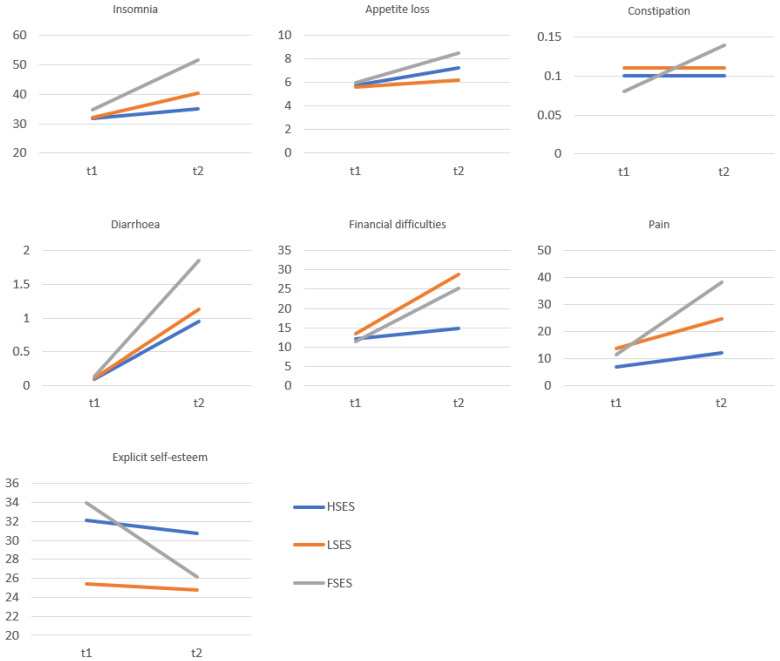
QoL (EORTC QLQ-C30) and explicit self-esteem (SES) before and after mastectomy among women low (LSES), fragile (FSES) or high (HSES) baseline self-esteem.

Taking account of groups differing in the type of self-esteem (low explicit and implicit self-esteem, fragile self-esteem and high explicit and implicit self-esteem), the largest decreases in various dimensions of QoL and explicit self-esteem were observed in women with fragile self-esteem (Figure 2). In groups with low and high self-esteem, the changes between the pre- and post-tests were much smaller. A group of women with high explicit and implicit self-esteem (HSES) showed the best profile of QoL parameters after mastectomy.

## 4. Discussion

Like Study 1, Study 2 showed that QoL and self-esteem deteriorated in breast cancer patients who underwent surgical removal of the tumour.

Simultaneous high explicit and implicit self-esteem proved to be conducive to the best functioning after the procedure. However, high, fragile self-esteem was associated with the greatest deterioration in both global self-esteem and QoL. This is consistent with the observation that fragile self-esteem is associated with the risk of mental state decompensation [41].

People with fragile self-esteem are often unaware of having conditional self-esteem [28]. Meanwhile, according to the concept of optimal self-esteem by Kernis [40], the mere knowledge of being dependent on external conditions—which is common in cancer patients, particularly those undergoing surgery—may pose a threat to high self-esteem.

Fragile self-esteem has its roots in the early and negative experiences of an individual, leading to low implicit self-esteem [55]. The self-esteem of these people is susceptible to information that may threaten them, and consequently these people take more defensive measures to maintain a high level of explicit self-esteem. This process can be suspected in patients in the preoperative period when they try to maintain high explicit self-esteem in response to the diagnosis and symptoms of cancer. Very often, fragile self-esteem also leads to a tendency to unrealistic optimism [55]. This factor may weaken the abovementioned defence mechanisms for self-esteem, and the negative effects of the excision of tissues affected by cancer reduce these defence mechanisms even more. It has also been shown that implicit self-esteem is particularly sensitive to affective factors [41,53,55], and these may take the form of an intensified negative affect after surgery and in the face of the consequences of this surgery.

Fragile self-esteem may also be associated with greater sensitivity to social factors, such as a sense of rejection, relational losses or unfair treatment [55]. This would explain the significant deterioration in QoL in the area of experiencing pain, shown in Study 2. Patients with fragile self-esteem reported the strongest pain two months after the procedure. Feelings of exclusion, loss or injustice are components of so-called social pain [56,57]. In this phenomenon, physical pain occurs in response to these social stimuli, which are common in cancer patients. Moreover, it has been shown that people with fragile self-esteem may be more likely to evaluate pain stimuli as more intense when their self-image is threatened [58].

Given the foundations of shaping fragile self-esteem, people who are in a situation of self-image threat—and this is what happens during surgical treatment of breast or oral cancer—will require a special approach to psychological support. It seems that therapeutic approaches aimed at the neurotic mechanisms of affect avoidance will be the most effective.

The results obtained in the studies may also suggest that preoperative self-esteem enhancement would be beneficial for the postoperative mental functioning of cancer patients. Psychotherapy offers the opportunity to improve self-esteem, but this process is time-consuming. Even in the case of short-term approaches, such as cognitive behavioural therapy, it takes at least several weeks. It means that these methods cannot be directly and fully used in patients awaiting surgery. However, it is possible to use selected techniques that can help protect self-esteem. For example, focusing on the patient’s strengths [59] and enhancing reflections that improve the patient’s agency [60], mindfulness [61] and/or thought diffusion techniques [62] are interventions that can open cognitive categories related to more positive thinking about oneself. On the other hand, ensuring a safe, respectful and warm atmosphere in the relationship with the patient may foster implicit self-esteem. In the latter case, it is essential to reinforce the appropriate behaviour of medical staff and relatives towards the sick person.

In the group of participants in both studies, the side effect of life-saving therapeutic procedures was the risk of bodily mutilation and deterioration of health-related quality of life. In our studies, there were no indications of medical malpractice, such as unnecessary excessive patient mutilation. However, it is worth emphasizing the importance of such a risk in future research and patient monitoring. The deterioration of the patient’s quality of life can lead to severe psychological effects, including depressive disorders and suicidal thoughts [63].

## 5. Limitations

The two studies presented indicate the important role of primary self-esteem and the surgical procedure in shaping the postoperative health-related quality of life. The first of the studies, conducted in a group of people exposed to easily noticeable changes in their appearance resulting from surgery in the oral cavity, has indicated changes in the quality of life depending on self-esteem. Importantly, it has been shown that high self-esteem is not unambiguously correlated with a higher quality of life after surgery. It is a premise for further research. This result is partly explained by the results of Study 2, which has shown that fragile self-esteem contributes to the deterioration of the quality of life after mastectomy. It indicates the need for further research on the change in the quality of life in cancer patients undergoing surgical procedures that carry the risk of bodily mutilation, taking into account the multidimensional approach to self-esteem. It is worth considering both explicit and implicit assessment, as well as expanding the exploration of the topic with multidimensional self-esteem scales. Here, the focus is solely on explicit global self-esteem. The studies presented analysed changes in the quality of life and self-esteem in people with various diagnoses and undergoing various surgeries, leading to diverse complications in body image (high heterogeneity). The test procedures used were also different. While in Study 1, all patients were hospitalised, Study 2 was conducted outside the hospital—in a laboratory at an academic centre. These different circumstances may have influenced other results obtained. Finally, Study 2 included only women with breast cancer, which makes it impossible to generalise the results to the entire population of cancer patients. In the future, it would be worth exploring the issue of overt and implicit self-esteem in a group of cancer patients undergoing surgery. However, increasing the sample size and exploring phenomena in groups with greater homogeneity will be beneficial. The impossibility of introducing the same research procedures, resulting from the specificity of treatment and side effects in both groups of patients, especially the inability to perform measurements at the same time intervals, means that the results of Study 1 and Study 2 cannot be directly compared. However, the results of these studies allow for a broader perspective and the possibility of a more comprehensive discussion.

## 6. Conclusions

Self-esteem is an important factor linked to QoL after surgical procedures leading to bodily deformities associated with cancer treatment. Our results suggest that high self-esteem is conducive to a better QoL and higher self-esteem after the procedure, provided that the individual also has high implicit self-esteem. Cancer patients with high, fragile self-esteem seem at risk of the greatest deterioration in QoL and self-image after cancer surgery. These people should be given special psycho-oncological care.

## Figures and Tables

**Table 1:** Characteristics of respondents surveyed in Study 1 (patients with oral cancer).

Characteristics		*n*	%
Gender	Male	15	42.9
	Female	20	57.1
Age	21–40 years	6	17.1
	41–60 years	15	42.9
	61–80 years	14	40.0
Place of residence	City	22	62.9
	Village	13	37.1
Education	Primary	5	14.3
	Vocational	9	25.7
	Secondary	7	20.0
	Higher	14	40.0
Diagnosis	Tongue	12	34.3
	Bottom of the mouth	6	17.1
	Gum	6	17.1
	Palate	5	14.3
	Lip	4	11.4
	Buccal mucosa	2	5.7

Notes: *n* = 35.

**Table 2:** Differences in QoL and self-esteem before and after surgery among patients with oral cancer.

QoL and Self-Esteem	Before Surgery		After Surgery		*p*	Cohen’s d
	M	SD	M	SD		
Global health status/QoL	64.05	16.27	38.10	22.44	<0.001	1.31
Physical functioning	84.00	17.28	66.10	26.88	<0.001	0.67
Role functioning	91.43	15.32	60.48	27.44	<0.001	1.22
Emotional functioning	54.76	27.06	53.57	25.35	0.807	0.04
Cognitive functioning	72.86	26.53	73.81	19.08	0.840	−0.03
Social functioning	62.38	32.42	57.14	25.97	0.299	0.18
Fatigue	22.86	22.05	50.48	30.17	<0.001	−0.75
Nausea and vomiting	1.43	4.73	16.67	24.25	<0.001	−0.60
Dyspnoea	5.71	15.09	20.00	25.82	0.002	−0.58
Insomnia	40.00	36.87	57.14	32.91	0.029	−0.38
Appetite loss	37.14	40.24	50.48	39.08	0.104	−0.28
Constipation	5.71	15.09	18.10	26.00	0.017	−0.42
Diarrhoea	0.95	5.63	2.86	9.47	0.324	−0.17
Financial difficulties	20.95	30.34	22.86	23.94	0.661	−0.07
Pain	19.76	20.32	43.33	19.99	<0.001	−0.93
Swallowing	7.38	13.67	45.48	28.25	<0.001	−1.13
Teeth	17.14	23.39	19.05	30.56	0.721	−0.06
Opening mouth	23.81	29.78	56.19	36.85	<0.001	−0.81
Dry mouth	24.76	24.71	60.00	26.57	<0.001	−1.13
Sticky saliva	30.48	26.04	56.19	28.89	<0.001	−0.69
Senses problems	2.86	8.56	16.19	20.80	<0.001	−0.71
Coughing	3.81	10.76	13.33	18.44	0.006	−0.50
Felt ill	23.81	29.78	41.90	31.67	0.002	−0.55
Speech problems	10.79	16.93	39.37	22.44	<0.001	−1.12
Trouble with social eating	18.33	20.29	47.62	26.86	<0.001	−1.02
Trouble with social contact	16.38	20.15	36.38	22.77	<0.001	−0.91
Less sexuality	36.19	39.29	54.76	40.94	0.016	−0.43
Pain killers	25.71	44.34	88.57	32.28	<0.001	−1.15
Nutritional supplements	11.43	32.28	45.71	50.54	0.002	−0.58
Feeding tube	0.00	0.00	65.71	48.16	<0.001	−1.36
Weight loss	28.57	45.83	57.14	50.21	0.010	−0.46
Weight gain	0.00	0.00	2.86	16.90	0.324	−0.17
Self-esteem	29.31	4.34	27.34	5.14	0.031	0.38

Notes: *n* = 35.

**Table 3:** Characteristics of respondents surveyed in Study 2 (breast cancer patients).

Characteristics		*n*	%	M	SD
Age				51.76	6.83
Education	Primary	4	4.2		
	Vocational	21	21.9		
	Secondary	35	36.5		
	High	36	37.5		
Marital status	Single	12	17.4		
	Married or living together	67	69.8		
	Divorced	11	11.5		
	Widowed	6	6.3		
Self-esteem	Explicit self-esteem			31.52	4.98
	Implicit self-esteem			0.42	0.63
	Low explicit and implicit self-esteem	21	21.9		
	High explicit and implicit self-esteem	32	33.3		
	Fragile self-esteem	43	44.8		

Notes: *n* = 96.

**Table 4:** Differences in QoL and global self-esteem before and after mastectomy among breast cancer patients.

QoL and Self-Esteem	Before Surgery		After Surgery		*p*	Cohen’s d
	M	SD	M	SD		
Global health status/QoL	78.71	15.42	68.42	20.67	<0.001	0.56
Physical functioning	96.18	16.01	89.96	21.15	<0.001	0.33
Role functioning	94.36	12.83	82.85	24.71	<0.001	0.58
Emotional functioning	59.97	23.95	47.11	21.63	<0.001	0.57
Cognitive functioning	92.92	24.66	92.76	21.04	0.890	0.01
Social functioning	77.87	30.27	67.23	22.75	<0.001	0.40
Fatigue	6.91	21.18	35.32	28.64	<0.001	1.13
Nausea and vomiting	0.39	3.36	1.13	5.51	0.562	0.16
Dyspnoea	3.61	11.92	2.55	14.77	0.713	0.08
Insomnia	33.24	32.70	43.76	31.03	0.002	0.33
Appetite loss	5.87	19.04	7.56	16.38	0.304	0.10
Constipation	0.09	4.13	0.12	9.56	0.875	0.01
Diarrhoea	0.12	3.07	1.40	8.89	0.504	0.19
Financial difficulties	12.19	18.22	22.55	13.94	<0.001	0.64
Pain	10.38	19.01	26.49	17.48	<0.001	0.88
Global self-esteem	31.52	4.98	27.36	5.02	<0.001	0.83

Notes: *n* = 96.

## Data Availability

The data presented in this study are available on request from the corresponding author.
